# Development of a Novel Task to Assess Fast and Slow Thinking in Schizophrenia

**DOI:** 10.1192/j.eurpsy.2025.2281

**Published:** 2025-08-26

**Authors:** B. Yıldırım, I. Gómez, P. Fuentes-Claramonte, M. Á. García-León, N. Ramiro, P. McKenna, E. Pomarol-Clotet

**Affiliations:** 1 FIDMAG Germanes Hospitalàries Research Foundation, CIBERSAM-ISCIII , Barcelona, Spain

## Abstract

**Introduction:**

Delusions in schizophrenia have been theoretically linked to probabilistic reasoning bias (‘jumping to conclusions’, JTC), although experimental support has been mixed (Garety *et al* BJP 2013; 203 327-333). Ward and Garety (Schiz Res 2019; 203 80-87) recently proposed a reformulation of the theory in terms of Kahneman’s concepts of ‘fast’ and ‘slow’ thinking. This proposes that decision-making involves two cognitive processes: a fast, heuristic-based approach which is prone to errors, and a slow, deliberate process that carefully evaluates all the relevant evidence. According to this view, an overreliance on fast thinking and/or reduced engagement of slow thinking underlies the initial development of delusional interpretations of everyday events and also makes them harder to be corrected.

**Objectives:**

Our aim was to develop a novel task to investigate the fast and slow thinking hypothesis of delusions in patients with schizophrenia, for use in behavioural and functional imaging studies. As a preliminary step, we tested this task on healthy participants.

**Methods:**

A battery of 137 experimental questions (where fast thinking leads to incorrect answers) was generated from multiple sources, including examples of the base rate and conjunction fallacies, the cognitive reflection test (CRT), trick questions, and syllogisms. Example questions included: If it takes 5 machines 5 minutes to make 5 widgets, how long would it take 100 machines to make 100 widgets? [Correct answer = 5 minutes; intuitive answer = 100 minutes; category: cognitive reflection] A farmer had 15 sheep and all but 8 died. How many are left? [intuitive answer: 7; correct answer: 8, category: trick question]. 137 control questions (where both fast and slow thinking give the correct answer) were adapted from the experimental questions. The questions were administered online to 176 healthy volunteers using PsychoPy software, with 15 experimental and 15 control questions randomly assigned to each participant.

**Results:**

The sample had a mean age of 40.3 years (range 17-77 years); 55.1% were female and 65.9% had a university education. Correct answers to experimental questions were markedly fewer than answers to control questions in all categories (overall p < 0.001). Response latency for the experimental questions was slightly higher than for the control questions, apart from in one category (CRT) (overall p = 0.004).

**Conclusions:**

Results from a large sample of healthy participants indicate that a battery of questions can be feasibly developed to reliably detect fast thinking.

**Disclosure of Interest:**

None Declared

